# Early Transcriptional Changes in the Midgut of *Ornithodoros moubata* after Feeding and Infection with *Borrelia duttonii*

**DOI:** 10.3390/microorganisms10030525

**Published:** 2022-02-28

**Authors:** Mandy Schäfer, Florian Pfaff, Dirk Höper, Cornelia Silaghi

**Affiliations:** 1 Institute of Infectology, Friedrich-Loeffler-Institut, 17493 Greifswald-Insel Riems, Germany; cornelia.silaghi@fli.de; 2 Institute of Diagnostic Virology, Friedrich-Loeffler-Institut, 17493 Greifswald-Insel Riems, Germany; florian.pfaff@fli.de (F.P.); dirk.hoeper@fli.de (D.H.)

**Keywords:** *Ornithodoros moubata*, *Borrelia duttonii*, midgut transcriptome, differential gene expression analysis, Argasidae, relapsing-fever spirochetes

## Abstract

Studies on tick-pathogen-host interactions are helping to identify candidates for vaccines against ticks and tick-borne diseases and to discover potent bioactive tick molecules. The tick midgut is the main tissue involved in blood feeding and, moreover, the first organ to have contact with pathogens ingested through the blood meal. As little is known about the molecular biology of feeding and tick defence mechanisms against microorganisms, but important for understanding vector-pathogen interactions, we explored the early transcriptional changes in the midgut of *Ornithodoros moubata* after feeding and in response to challenge with the relapsing-fever spirochete *Borrelia duttonii* using the Ion S5XL platform. Besides transcripts with metabolic function and immune-related transcripts we discovered numerous putative and uncharacterized protein sequences. Overall, our analyses support previous studies and provides a valuable reference database for further functional proteomic analysis of midgut proteins of *O. moubata*.

## 1. Introduction

Numerous emerging infectious diseases are caused by zoonotic pathogens, including those transmitted by hematophagous arthropod vectors such as mosquitoes and ticks. Ticks and tick-borne diseases affect animal and human health worldwide and are often of great economic significance [1]. As tick control in livestock has to cope acaricide resistance and concerns are raised regarding the environmental impact of pesticides, new approaches for controlling ticks and preventing the transmission of pathogens are focusing on the molecular interactions between host, vector and pathogen. The aim is to identify promising candidates for vaccines against ticks and tick-borne diseases by reducing the tick infestation and preventing ticks from transmitting disease-causing pathogens [2].

In the late 1990s, a protective concealed antigen, the glycoprotein Bm86, was characterized from the midgut of the bovine tick *Rhipicephalus microplus* [3]. Based on the recombinant Bm86 antigen, the first and so far, only vaccine that have made it to the market was developed, which mainly reduce the average fertility of adult ticks due to a disturbed blood uptake [4]. Since then, intensive basic research focused on transcriptome and proteome profiling in tick salivary glands and midgut, as these structures represent key interfaces between host, vector and pathogen [5]. In a recent article, Tirloni et al. illustrate the dynamic changes in gene expression in embryos, ovaries, salivary glands, fat body and digestive cells during the parasitic phase and the embryonic development of *R. microplus*. The study reveals over 10,000 novel coding sequences that have highly tissue-specific expression profiles giving insights into the physiology of production and role of saliva, blood digestion, energy metabolism and development [6]. Beside organ-specific analysis of gene expression, special attention is paid to expression differences between female and male ticks as females are the key component of the tick life cycle and represent a suitable target for vaccine development. Oleaga et al. used two different mass spectrometry approaches to explore the sialoproteome of adult *Ornithodoros moubata* ticks. Quantification of salivary proteins by SWATH-MS indicated significant quantitative differences in the salivary proteome between male and female ticks, which should be considered when selecting vaccine targets, as suggested by the author [7]. Follow-up studies identified several major protein families that play important roles in tick development, feeding and digestion, host attachment and specificity, resistance to acaricides and response to abiotic factors [8,9]. Some of these proteins have recently been selected and their individual and joint vaccine efficacy were explored as protective candidate antigen in ixodid and argasid ticks [2,10,11]. However, no other anti-tick vaccine, except the Bm86-based one, have been commercialized so far due to different challenges discussed by de la Fuente and Estrada-Peña [12].

A positive side effect of using ‘omics technologies is the discovery of possibly bioactive molecules that are seen as promising source of novel therapeutics [13]. Tick saliva in particular, a complex cocktail of molecules that ticks inject into their hosts to inhibit pain, prevent blood clotting and suppress the host immune system, has potential for medical exploitation [14]. Several protein families with immunomodulatory activity, such as evasins, serpins, cystatins and lipocalins have been identified in ticks. Evasins, for example, are a class of cysteine-rich chemokine-binding proteins that modulate the host inflammatory response by binding to host chemokines. Francka et al. have shown, using the evasin ACA-01 from *Amblyomma cajennense*, that posttranslational modification trough sulfation can modulate binding affinity for proinflammatory chemokines and enhanced the ability to inhibit chemokine signaling through cognate receptors and may provide a strategy to optimize tick salivary proteins for anti-inflammatory applications [15]. However, the development of bioactive molecules into therapeutics is still in its infancy due to lack of follow-up studies and the high cost of drug development. Furthermore, the efficacy of proteins is based on a synergistic effect on compounds from a complex mixture, which is not captured by studies of individual genes or proteins [13].

From the medical and veterinary perspective hard ticks (Ixodidae) represent the most important tick family. They are responsible for transmitting a variety of viral, bacterial and protozoan agents of disease while taking a blood meal from their host. Some hard ticks can also elicit tick paralysis, caused by a salivary toxin released while feeding [1]. Hence it is not surprising that many studies are focusing on the family Ixodidae. Argasid ticks receive less attention despite their role as vectors and reservoirs of pathogens. *Ornithodoros moubata* is widespread throughout southern and eastern African countries [16]. This tick species is the main vector of the East African human relapsing fever causing agent *Borrelia duttonii* [17] and of African swine fever virus in pigs [18]. It has also been identified as competent vector for *Coxiella burnetii* [19] and West Nile virus [20].

Compared to hard ticks, transcriptome [21,22,23,24,25,26,27,28] and proteome profiling [21,29,30,31,32] have been performed on a small number of soft tick species only, with only a few focusing on the midgut. The midgut embodies the primary organ for tick survival since it is responsible for blood digestion and absorption of nutrients [33]. Furthermore, the midgut is the first tissue where pathogens ingested with the blood meal are exposed to the tick’s internal tissues although there are not in direct contact with digestive enzymes as digestion in ticks takes place intracellular [34]. Oleaga et al. compared the midgut transcriptome of *O. erraticus* and *O. moubata* female ticks before and after a blood meal and identified genes whose expression are differentially regulated after feeding [23,25]. They provided novel information’s on argasid genes and proteins involved in the processes of blood digestion, nutrient transport and metabolism and response to the oxidative stress that is produced during blood digestion. Similar results were reported by Landulfo et al. where gut transcriptome analysis revealed several transcripts associated with hemoglobin digestion, such as serine, cysteine and aspartic proteases and metalloenzymes [24]. Phylogenetic analysis of digestive peptidases using transcriptomic data confirmed that most of them are clustered with cathepsin peptidases of other tick species. The midgut transcriptome profile of *O. rostratus* fourth instar nymphs described by Araujo et al. revealed that Defensin A and Ferritin were the most abundant transcripts represented ~17% of all RNA expressed in the midgut tissue [26]. However, transcriptomic studies exploring the tick immunity in response to pathogen infection are also limited to hard ticks (*Amblyomma*, *Ixodes*, *Rhipicephalus*) and focusing to a few but significant pathogens such as Lyme *Borrelia burgdorferi* [35] and *Borrelia afzelii* [36], *Anaplasma* spp. [37,38,39] and *Rickettsia rickettsii* [40].

In this study we want to characterize the early transcriptional changes in the midgut of *O. moubata* in the unfed, engorged and *Borrelia duttonii*-infected state. The aim is to identify transcripts that are differentially expressed in the respective physiological state and therefore play a key role. Our intention is to contribute to the understanding of the molecular mechanisms, in particular the molecular interaction between soft ticks and relapsing fever borrelia, as this knowledge forms the basis for the development of control strategies in general. To our knowledge, this is the first report of differential expression of midgut transcripts in response to infection with a relapsing fever borrelia in *O. moubata*.

## 2. Materials and Methods

### 2.1. Ticks Rearing and In Vitro Feeding

Colonies of *O. moubata* were maintained in the insectary within the biosafety level 2 facilities. The ticks were originally received in 2015 from the Department of Veterinary Medicine at the Freie Universität Berlin, where they were maintained over many years. All developmental stages were kept in darkness in an incubator (25 °C; 85% RH) within *Drosophila* cultivation tubes (Carl Roth, Karlsruhe, Germany) lined with filter paper to absorb coxal fluid and excretory waste. An artificial feeding system was applied to fed the ticks in vitro [41]. Therefore, a feeding chamber is placed on a heating plate adjusted to 36–38 °C, filled with fresh defibrinated pig blood obtained from a commercial source (LOT 35022500/01; Fiebig Nährstofftechnik, Düsseldorf, Germany) and stirred at 700 rpm to simulate a natural pulse. The ticks are placed in a second feeding chamber set on top, which is stretched with a butyric acid-treated Parafilm membrane. Ticks were allowed to feed until they were replete and had detached themselves from the membrane. Subsequently the fed ticks were incubated under the conditions outlined above to enable moulting and oviposition.

### 2.2. Experimental Setup and Tissue Collection

To address the question of differentially regulated genes through blood feeding and spirochetal infection, midgut samples of female *O. moubata* that were either unfed, engorged to repletion and infected with *Borrelia duttonii* were prepared. For each physiological condition, three biological replicates, represented each by single individuals, were processed. To obtain non-infected unfed females, unfed adult *O. moubata* which had not fed for six months were used. To obtain non-infected engorged females, unfed adult *O. moubata* were allowed to feed to repletion as described above. Swine blood of the same lot number was used to obtain *B. duttonii*-infected females, except that the blood was spiked with in vitro cultured *B. duttonii* at a final concentration of 2 × 10^6^ spirochetes/mL, which corresponds to the spirochaetemia in the blood of an infected BALB/c laboratory mice during the first few days of infection [42]. Spirochetes were purchased from the German National Reference Center for Borrelia and cultured in Barbour-Stoenner-Kelly medium (BSK-H) containing 6% heat-inactivated rabbit serum under standard conditions. The species was confirmed by 16S rRNA gene sequencing. Tick midguts were dissected from unfed females and from fed females 24 h post feeding. Therefore, ticks were briefly washed with sterile phosphate buffer saline (PBS) before dissections. Tick dorsal integuments were cut with a surgical spring scissor and the body cavity was rinsed with PBS for better visualization. Midguts were then removed with forceps and washed briefly in PBS to remove contaminants.

### 2.3. RNA Extraction, Library Preparation and Sequencing

Washed midguts were snap frozen in liquid nitrogen and cryofractured individually using the cryoPREP impactor (Covaris, Brighton, UK). Total RNA was isolated from the pulverized midgut by adding 750 µL TRIzol LS Reagent (Life Technologies, Darmstadt, Germany) and 250 µL chloroform. After centrifugation at 13,000× *g* (4 °C) the upper aqueous phase was collected and used as input for the Agencourt RNAdvance Tissue Kit (Beckman Coulter, Krefeld, Germany) in combination with a KingFisher Flex System (Thermo Fisher Scientific, Darmstadt, Germany) for automated extraction. A DNAse I (Qiagen, Hilden, Germany) digestion was included prior to final elution. After that, an appropriate amount of ERCC spike-in mix (Life Technologies, Carlsbad, CA, USA) was combined with 2 µg total RNA for extraction of the poly(A) RNA fraction using the Dynabeads mRNA DIRECT Kit (Invitrogen, Carlsbad, CA, USA) according to the manufacturer’s instructions. Fragmentation and construction of the strand specific whole transcriptome library was done using the Ion Total RNA-Seq Kit v2.0 (Life Technologies, Carlsbad, CA, USA). The quality and integrity of the RNA, cDNA and final library were verified using the Bioanalyzer 2100 (Agilent Technologies, Santa Clara, CA, USA) and appropriate chips and reagents. The final libraries were quantified using the KAPA Library Quantification Kit (Kapa Biosystems, Cape Town, South Africa) in combination with a CFX96 Real-Time PCR Detection System (Bio-Rad Laboratories, Feldkirchen, Germany). Subsequently, equimolar amounts of the library were then sequenced using an Ion S5XL system in combination with an Ion 540 chip (Life Technologies, Carlsbad, CA, USA) along with appropriate sequencing reagents according to the manufactures instructions.

### 2.4. De Novo Assembly of O. moubata Midgut Transcriptome

Raw Ion Torrent derived reads were initially quality trimmed and remaining specific adapter sequences as well as host specific rRNA sequences (reference: MF818022.1) were removed using the 454 Sequencing System Software (version 3.0; Roche). The trimmed reads from all biological replicates were combined and then used for a de novo assembly using SPAdes (version 3.15.3; [43]) running in Ion Torrent compatibility mode “--iontorrent” and SPAdes option “--rna” for RNA-Seq data.

Additionally, we received all publicly available Short Read Archive (SRA) datasets for the species *O. moubata* and individually trimmed them with trim-galore (version 0.6.7) and Cutadapt (version 3.5) using a quality phred score cutoff of 20 and adapter auto-detection. The combined and trimmed SRA datasets were then assembled using SPAdes (version 3.15.3) running in paired-end mode with option “--rna”. Subsequently, the de novo assembled transcript sequences from both, the Ion Torrent raw data obtained in this study and from the SRA, respectively, were combined. The quality of the individual de novo assemblies was benchmarked using BUSCO (version 5.2.2) along with the “*arachnida_odb10*” lineage dataset (creation date: 5 August 2020, number of genomes: 10, number of BUSCOs: 2934). In order to characterize the transcripts, a custom protein reference was created (n = 854,613 entries). In detail, manually reviewed (Swiss-Prot) and unreviewed entries (TrEMBL) of the UniProt Knowledgebase (https://www.uniprot.org/uniprot; accessed on 16 November 2021) were downloaded. Only TrEMBL entries belonging to the subphylum Chelicerata (NCBI taxonomy ID 6843) were accessed. The Swiss-Prot and TrEMBL protein sequences were clustered using cd-hit (version 4.7; [44]) with default options and a blast database was created. In order to characterize the transcripts, we then matched the de novo sequences to the clustered protein references using blastx (version 2.12.0+) with a cutoff E-value of 0.001.

### 2.5. Differential Expression Analysis of the Midgut Transcriptome

In order to quantify the expression of the RNA transcripts within each biological replicate of the different treatment groups the software Salmon (version 0.14.1; [45]) was used. Initially, a salmon index was created from the de novo assembled and filtered transcripts using a perfect hash and a k-mer size of 31. Subsequently, the trimmed reads were used for quantification using quasi-mapping mode and options for sequence-specific and fragment GC bias correction, alignment-based verification of mappings and 100 bootstrap samples per library. A tx2gene table was created using the annotation from the blastx analysis and the quantification results were further analyzed using DESeq2 (version 1.26.0; [46]) as available for R (version 3.6.2; https://www.r-project.org, accessed on 16 November 2021) in combination with RStudio (version 1.2.5033; https://www.rstudio.com, accessed on 16 November 2021). For initial data exploration, the read counts were normalized using the regularized log transformation (function rlog) and a principal components analysis was conducted (function plotPCA) using only the 2000 most variable transcripts. The dataset was tested for differential transcript expression (function dds) between the states “engorged vs. unfed—non-infected”, as well as “*Borrelia duttonii*-infected vs. non-infected—engorged”, using an adjusted *p*-value of 0.05 and a log2 foldchange of |1| as cutoffs. Log2 foldchanges were shrinked (function lfcShrink) prior to filtering and ranking using the “apeglm” algorithm [47]. Differentially expressed transcripts were subsequently categorized into GO terms using the UniProtKB Retrieve/ID mapping tool.

## 3. Results

### 3.1. De Novo Transcriptome Assembly and Functional Annotation of O. moubata Transcriptome

From a total of about 92 million Ion Torrent derived single-end reads from nine *O. moubata* midgut samples representing three biological treatments a de novo transcriptome was assembled. This transcriptome comprised 15,083 sequences with an average length of 1065.2 bp, a N50 of 1213 bp and a maximum contig length of 5729 bp (Table 1 and Table S1). Additionally, we used roughly 334.5 million Illumina derived paired-end reads from the SRA that represented samples from salivary glands of *O. moubata* under three different feeding conditions (n = 6) and samples from the *O. moubata* midgut after feeding (n = 2) (see Table S2). This de novo assembly resulted in 202,950 sequences with an average length of 1943.5 bp, a N50 of 5169 bp and a maximum contig length of 56,176 bp. A combined transcriptome using Ion Torrent derived reads from this study along with SRA derived data simultaneously was not possible, as this is explicitly not recommended by SPAdes developers. Therefore, we decided to combine both assemblies by simple merging them. We did not apply any clustering to reduce redundancy as read counts for duplicated transcripts will be collapsed based on the blastx results.

**Figure 1:**
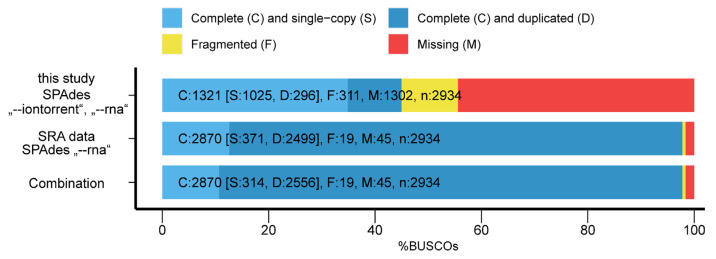
BUSCO assessment results for the de novo transcriptomes.

The BUSCO analysis showed that of the 2934 BUSCO groups searched, only 45.0% were detected and 44.4% were missing (Figure 1). Within the assembly from the SRA derived data BUSCO detected 97.8% of the BUSCO groups while 1.6% were missing. The combined transcriptome showed comparable results to SRA derived data with slightly higher duplication rate.

### 3.2. Differential Expression Analysis of the Midgut Transcriptome

**Figure 2:**
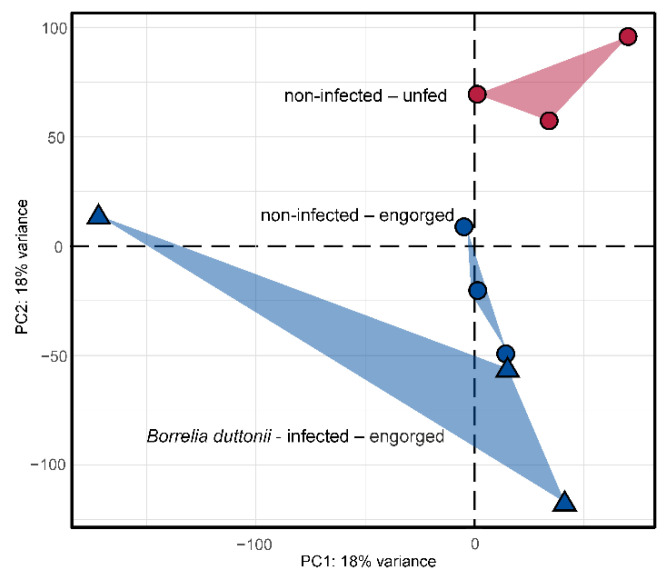
Principle components analysis for a subset of 2000 transcripts showing the highest variance within the dataset. Read counts were normalized using the regularized log transformation prior to analysis. Samples from non-infected and *Borrelia duttonii*-infected samples are highlighted with circular or triangle shapes, respectively. Samples from *O. moubata* ticks that have either been allowed to fed to repletion or unfed are colored in blue and red, respectively.

Initially, the overall variance within the dataset was inspected using a principal component analysis (Figure 2). Samples of the same group should maintain closest distance, while samples from different groups should be separated. Samples from the “non-infected—unfed” and “non-infected—engorged” groups clustered well together, while samples from the “*Borrelia duttonii*-infected—engorged” group were more spread. However, samples from both non-infected groups were clearly separated from each other, indicating differences based on starvation. Samples from the “*Borrelia duttonii*-infected” group were very diverse but different from the other samples.

The gene expression levels were then compared between two physiological states in more detail: “engorged (n = 3) vs. unfed (n = 3)—non-infected” and “*Borrelia duttonii*-infected (n = 3) vs. non-infected (n = 3)—engorged”. Cut off values were set to 0.05 for the adjusted *p*-value and >1 for the absolute shrinked log2 foldchange.

**Figure 3:**
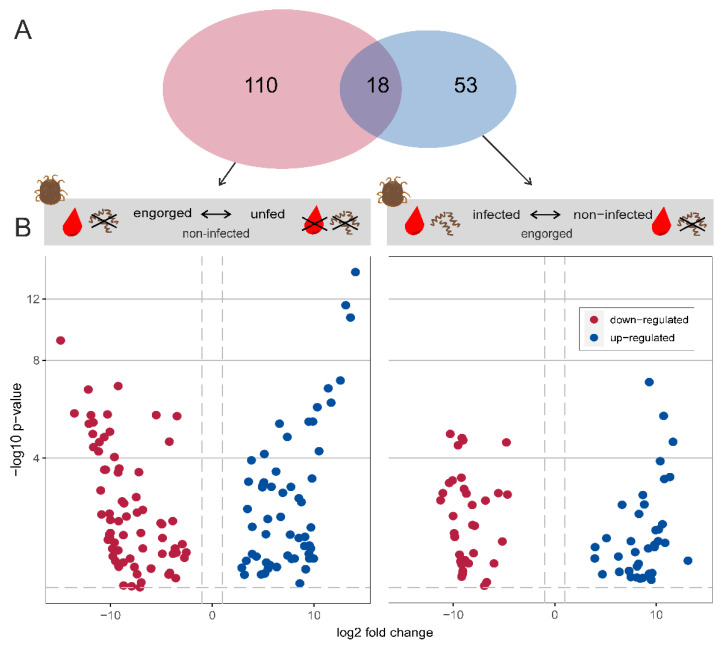
Differential expression analysis. (**A**) Venn diagram showing the total number of differentially expressed transcripts in each experimental group. (**B**) Volcano plot showing differentially expressed transcripts between the states “engorged vs. unfed—non-infected” and “*Borrelia duttonii*-infected vs. non-infected—engorged”. Blue and red dots represent up- and down-regulated transcripts, respectively. The dashed lines indicate the used thresholds of 0.05 for adjusted *p*-value and |1| for log2 foldchange.

A total of 128 transcripts were significantly differentially expressed when comparing “engorged vs. unfed—non-infected” *O. moubata* ticks (71 and 57 are down- or up-regulated, respectively) (Table S3, Figure 3). Of the 128 transcripts, 108 transcripts were mapped to UniProtKB IDs with or without GO terms, of which 57 are down-regulated and 51 are up-regulated. When comparing “*Borrelia duttonii*-infected vs. non-infected—engorged” *O. moubata* ticks, 71 transcripts were significantly differentially expressed (34 and 37 are down- or up-regulated, respectively) (Table S3, Figure 3). Of the 71 transcripts, 59 were mapped to UniProtKB IDs with or without GO terms, of which 27 are downregulated and 32 are up-regulated. In total, 181 transcripts were differentially expressed, including 18 transcripts shared by both groups (Table S3).

**Figure 4:**
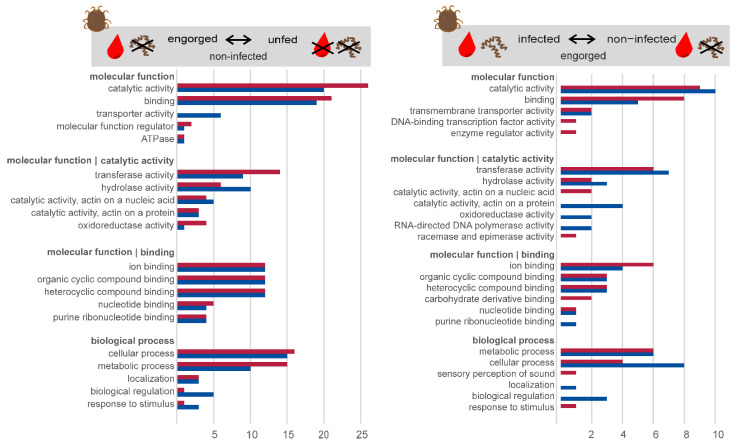
Classification of differentially expressed transcripts. Gene ontology annotation of up-regulated (red) and down-regulated (blue) transcripts in the midgut of *O. moubata* in the comparison “engorged vs. unfed—non-infected”, and “*Borrelia duttonii*-infected vs. non-infected—engorged”. The categories molecular function and biological process are shown.

The comparison with the user-defined protein reference, including 854,613 protein sequences, made it possible to annotate 150 of the 181 differentially expressed transcripts. The remaining 31 transcripts showed no significant matches with other sequences within the databases searched, suggesting that many of the differentially expressed transcripts are previously unknown. Possible reasons for the lack of sequence homology can be artefacts of the assembly process, poor assembly quality, taxonomic isolation of the species and the resulting lack of data. The formation of new genes through duplication and/or transposition mechanisms has already been described [48,49]. The blastx annotated sequences were next functionally characterized using the information available in the Gene Ontology (GO) database and classified according to their molecular function and biological process. Figure 4 shows the classification of differentially expressed transcripts in the midgut of *O. moubata* in the analysis of “engorged vs. unfed—non-infected” and “*Borrelia duttonii*-infected vs. non-infected—engorged”. In the molecular Function category of “engorged vs. unfed—non-infected”, the most commonly annotated functions were catalytic activity (n = 46) and binding activity (n = 40), which is consistent with results observed in the midgut transcriptome of *O. mimon* [24], *O. moubata* [23] and *O. erraticus* [25]. Similar results were also documented by other authors in hard ticks [50,51,52,53]. The remaining annotated functions were divided as follows: transporter activity (n = 6), molecular function regulator (n = 3) and ATPase (n = 2). Similarly, the classification of the transcripts according to biological processes resulted in five categories. The following, more frequently occurring categories corresponded to transcripts that were involved in cellular processes (n = 31) and metabolic processes (n = 25). The remaining functional annotations were less numerous, the most common being biological regulation (n = 6), localization (n = 6) and response to stimulus (n = 4).

The classification of differentially expressed transcripts in the midgut of *O. moubata* in the analysis “*Borrelia duttonii*-infected vs. non-infected—engorged” shows that only about half as many transcripts are differentially regulated (n = 71). In the molecular function category, the most frequently annotated functions were catalytic activity (n = 19) and binding activity (n = 13). The remaining annotated functions were divided as follows: transmembrane transporter activity (n = 4), DNA-binding transcription factor activity (n = 1) and enzyme regulator activity (n = 1). Similarly, classification of transcripts by biological processes resulted in seven categories. The following more frequent categories corresponded to transcripts involved in cellular processes (n = 12), metabolic processes (n = 12) and biological regulation (n = 3). The remaining functional annotations were less numerous and were divided into four categories, the most frequent being involved in localization (n = 1), sensory perception of sound (n = 1), homeostatic process (n = 1) and response to stimulus (n = 1). 

**Figure 5:**
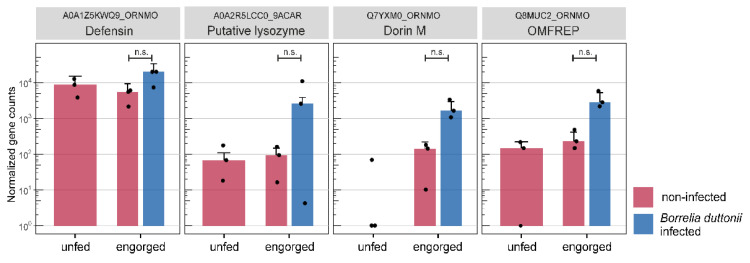
Immune related transcripts. Boxplots show the normalized gene counts of transcripts that have been related to tick immune response. The different sample groups are highlighted by color: unfed (red), engorged (red) and *Borrelia duttonii*-infected (blue). Error bars represent the standard deviation and dots represent individual samples; ns: not significant.

Some selected transcripts, possibly related to feeding and digestion of the blood meal, were examined in more detail after annotation and functional characterization with respect to assignment to specific enzyme classes. Table 2 shows transcripts that are related to the digestion of the blood meal and nutrient uptake. These are mainly oxidoreductases, transferases and hydrolases. Furthermore, among the 59 differentially expressed and annotated transcripts from the analysis of “*Borrelia duttonii*-infected vs. non-infected—engorged”, we sought to identify those transcripts that might be differentially regulated in response to infection *with B. duttonii*. As no distinctly immune-related transcripts were significantly differentially expressed, we then searched our assembly for non-differentially expressed immune-related transcripts. This was done by comparison with a user-defined protein reference (n = 1535) extracted from UniProtKB using the organism group “Acari” and the following gene ontology terms: defence, dorin, lysozyme, oxidative stress response, antimicrobial peptide, innate immune response. Out of 1535, 72 proteins are included in our assembly and their expression levels in the midgut of unfed, engorged and *B. duttonii*-infected *O. moubata* groups are shown in Figure S1. Among these protein matches, four transcripts, namely defensin, putative lysozyme, Dorin M and OMFREP, showed notably higher but non-significant expression levels in the *B. duttonii*-infected group (Figure 5).

## 4. Discussion and Conclusions

Differential expression analysis is used to detect quantitative changes between experimental groups or conditions and to identify biologically significant differentially expressed genes. Here, we analyzed differentially expressed midgut-specific genes from unfed, engorged and *Borrelia dutonii*-infected *O. moubata* and further characterized genes involved in the metabolism of nutrients and in defense response and response to oxidative stress soon after feeding and infection with the relapsing fever spirochete *B. duttonii*.

### 4.1. Tick Metabolism

The differences in the feeding, mating and oviposition strategies of the two families Argasidae and Ixodidae are reflected in the process of digestion. While digestion in argasid ticks begins after the tick dropped off the host, the processes of feeding and digestion in ixodid ticks are not separated in time. Digestion in female argasid ticks is also dependent on mating status, which may occur before or after feeding, with no differences in the amount of blood ingested. While mated females digest the blood meal, develop eggs, oviposit and then are ready to repeat the gonotrophic cycle, virgin females may store undigested haemoglobin as a nutrient reserve until mating occurs [54]. By contrast, hard ticks feed only once per life-stage and females die several days after oviposition. What both families have in common, however, is that digestion takes place in the acidic intracellular compartments of the intestinal epithelium [55], unlike in blood-feeding insects, which feed in the neutral pH of the intestinal lumen and digest blood rapidly [56].

By analysing the mialome of *O. moubata* we have identified 128 transcripts that are differentially expressed between “unfed” and “engorged” *O. moubata*, suggesting that they are probably involved in protein digestion, carbohydrate metabolism and transport, lipid metabolism and transport and in response to oxidative stress associated with blood feeding (Table 2). Of these, 108 (84.4%) transcripts were annotated and the remaining 20 (15.6%) transcripts were classified as unknown as they did not yield any hits within the searched database. This leads to the assumption that these (unknown) transcripts reflect (i) genes that have not been transcribed in the few soft tick studies conducted so far and are therefore still undiscovered. For example, Oleaga et al. [23] analysed the mialome of unfed *O. moubata* females and that of fed females 48 h post-feeding, versus 24 h post-feeding in our study, resulting in a different gene expression profile; (ii) genes that correspond to new genes that are unique to the *O. moubata* strain used due to many years of inbreeding. Charrier et al. [57] studied polymorphism in a highly inbred laboratory strain of *Ixodes ricinus* and showed that the laboratory strains have less heterozygosity and differ strongly from wild ticks. In this context it is interesting to mention that Pereira De Oliveira et al. observed higher susceptibility for ASFV infection in a long-time lab-reared colony of *O. moubata* compared to field collected *Ornithodoros* sp. [58]. They hypothesized that lab-reared ticks are highly inbred with fixed alleles and have a poor microbiome, resulting in a poorly stimulated or even immunologically naïve immune system compared to ticks collected from the field; (iii) represent non-coding RNA (ncRNA) sequences. Non-coding RNAs are known to participate in vector-parasite-host-interaction, either by modulate vertebrate host responses and favour vector survival or to be regulated in the host by parasites to favour parasite survival [59]. Many other studies on both, hard and soft ticks, also described ncRNAs in transcriptomic studies, but their regulatory functions in the processes of blood digestion is unknown [23,50].

In contrast to previously published studies, the number of differentially expressed genes in our study is significantly lower. This can be explained by biological reasons due to no altered expression level in “unfed” and “engorged” condition, or by technical reason due to conservative filtering approaches on RNA-seq data. As shown in the immune-related transcripts (Figure 5), interindividual differences in gene expression can also prevent a statistically significant result. As expected, the most important categories comprised transcripts for the common ‘housekeeping’ genes, i.e., oxidoreductases, hydrolases and transferases. At least one transcript showed identity with a metalloaminopeptidase from UniProtKB entries of *O. moubata* (A0A1Z5KYM0). In general, metalloproteases have been found in various tick species, including hard ticks and soft ticks and are among the most abundant type of proteases in ticks, which have a broad spectrum of activity that includes fibrinogenolysis [60] and blood protein digestion [61]. Most of them have been described in the salivary glands/saliva, where they are thought to play an important role in inflammation, immunomodulation, fibrinolysis, blood protein digestion, nociception, vitellogenesis, remodelling of extracellular matrix and pathogen transmission [62]. Oleaga et al. identified several transcripts with metallopeptidase activity in the midgut of *O. erraticus* and *O. moubata* by proteomic and transcriptomic approach which likely involved in haemoglobin and albumin digestion [23,31]. A recent study, Perner et al. identified zinc-dependent metalloproteases as the predominant salivary immunogens and showed that neutralisation of tick Zn-dependent metalloproteases by rabbit immunoglobulins or inhibition by phosphoramidon prevented ticks from initiating and continuing natural feeding, but not in vitro on an artificial membrane system [63]. They conclude that metalloproteases present in tick saliva target the components of the host hemostatic and defence systems that are absent during artificial membrane feeding.

A transcript (A0A293LQ15) up-regulated in the process of blood digestion was functionally annotated as aspartic-type endopeptidase. These enzymes are involved in the proteolytic pathway of hemoglobin degradation in the digestive vesicles in the midgut cells of hard ticks [64]. Since proteins are the largest and most important content in vertebrate blood, transcripts with lipid and carbohydrate transport and metabolism are differentially expressed in lower numbers. Furthermore, Grandjean et al. describes that lipids and carbohydrates are detectable in the cells two days after the blood meal and can also be stored within the cell [65,66]. Accordingly, the expression of genes involved in the metabolism of carbohydrates and lipids after blood feeding could start at a later stage or maintain a constant level of expression regardless of nutritional status. A transcript which was up-regulated in the course of *Borrelia duttonii*-infection was functionally annotated as putative chitinase (A0A2R5LGB3). In many blood-feeding arthropods, such as mosquitoes and ticks, the midgut is covered by a peritrophic membrane (PM), primarily composed of chitin, along with glycoproteins and other polycarbohydrates. Beside its function as a structural barrier, the PM can play a critical role in the arthropod innate immune defense against invading pathogens [67]. In *Ixodes scapularis*, it was shown that the disruption of the PM affect the persistence of *Borrelia burgdorferi* spirochetes in the gut [68]. On the other hand, parasites such as *Plasmodium* and *Leishmania* are known to secrete chitinases that enable their movement and also aid their dissemination through the gut [69,70]. In *O. moubata* and *O. erraticus*, enzymes have recently been identified that are thought to be involved in chitin metabolism and in the formation and maintenance of the peritrophic membrane during the digestive process [23,25].

The haemoglobin digestion releases a significant amount of haem and iron, which imbalance the production of reactive oxygen species and antioxidant defences, known as oxidative stress [71]. In ticks, excessive haem is detoxified by aggregation in specialised organelles in the midgut epithelium termed haemosomes [72]. In the midgut transcriptome, we identified up to 10 antioxidants and proteins involved in detoxification processes. Half of which were either up- or down-regulated after feeding which support to maintain a redox homeostasis in the cells. Interestingly, the transcript Cytochrome P450 (A0A1Z5LEL5) was differently expressed in both experimental groups. In the “engorged vs. unfed—non-infected” analysis, Cytochrome P450 was up-regulated after feeding, whereas in the “*Borrelia duttonii*-infected vs. non-infected—engorged” analysis it was down-regulated in the *B. duttonii*-infected group. Cytochrome P450s are members of a family of genes whose expression levels can rapidly change in response to external environmental stresses [51]. Cytochrome P450 enzymes have already been identified in saliva of *O. moubata*, *Amblyomma maculatum*, *I. ricinus* [30,73,74] and in ixodid ticks after bacterial and fungal challenge [51,75]. Thus, the P450s are also assumed to participate in detoxification during the infection response process in the tick gut [51]. Beside the heme binding proteins, we identified enzymes involved in the glutathione catabolic process and proteins with sulfotransferase activity. Putative gamma-glutamyltransferase are present in cell membranes and involved in cross cell membrane trafficking of amino acids and peptides as well as glutathione metabolism [76]. Glutathione peroxidases are antioxidant enzymes that catalyse the glutathione-dependent reduction of organic peroxides and hydrogen peroxide and are part of the cellular defence against the effects of oxidative stress. In our study, the expression of glutathione peroxidase is about 12 times higher in fed ticks than in the unfed state and down-regulated in the *Borrelia duttonii*-infected group compared to the fed group. Bourret et al. documented higher expression values in the salivary glands of *Ornithodoros turicata* infected with *Borrelia turicatae* [28]. The upregulation of such oxidases upon infection may represent an attempt by ticks to control pathogens by producing hydrogen peroxide. Narasimhan et al. showed that salp25D, a glutathione peroxidase homologue in the salivary glands of *Ixodes scapularis*, detoxified reactive oxygen species at the vector-pathogen-host interface, thereby providing a survival advantage to *B. burgdorferi* at the tick feeding site in mice [77]. However, what direct or indirect influence the redox balance has on *B. duttonii* infection in *O. moubata* midgut is unknown. It is also possible that the microbiome balance in the midgut is affected, which again may have an influence on pathogen transmission.

### 4.2. Tick Immune System

In contrast to the expression patterns in the analysis of “engorged vs. unfed—non-infected”, only half as many transcripts (n = 71) were significantly differentially expressed in the analysis “*Borrelia duttonii*-infected vs. non-infected—engorged”, of which 34 transcripts are down-regulated and 37 transcripts are up-regulated. Of these, 59 (83.1%) transcripts were annotated and the remaining 12 (16.9%) transcripts were classified as unknown as they did not yield any hits within the searched database. According to the GO annotation, many transcripts are assigned to be integral components of membranes. Integral membrane proteins have a range of important functions, including channelling or transporting molecules across the membrane or acting as cell receptors. As no distinctly immune-related transcripts were significantly differentially expressed, we then searched our assembly for non-differentially expressed immune-related transcripts and identified four transcripts which are slightly up-regulated after infection with *Borrelia duttonii*. Among these transcripts, two transcripts were annotated as lectins (Q8MUC2, Q7YXM0). It is known that lectins in ticks are involved in the humoral immune response by non-specific pathogen-recognizing defence systems [78]. Dorin M was the first lectin purified from the haemolymph of the soft tick *O. moubata* and later classified as a member of the fibrinogen-related protein family [79]. This lectin shows preferential binding specificity for sialic acid, N-acetyl-d-hexosamines and sialoglycoproteins [80]. Since sialic acid has been identified as a constituent of bacterial cell walls including the spirochete *Borrelia burgdorferi*, the authors assume that the plasma lectin dorin M may also take part in mechanisms of transmission of other spirochetes including *B. duttonii*. Beside dorin M, two more lectins (OmGalec, OMFREP) have been described in the haemocytes, midgut, ovaries and salivary glands of soft ticks of the genus *Ornithodoros* [81]. Studies characterising lectins in ticks of the genus *Ixodes* have shown that so-called ixoderins are important for the phagocytosis of Gram-negative bacteria and yeasts by haemocytes in the haemolymph and that their expression is up-regulated after injection of microorganisms, wounding or after blood ingestion [82]. This suggests that lectins in ticks are crucial immune proteins that cause phagocytosis or lysis of microorganisms and that these proteins generally up-regulated in the course of feeding on a potentially infected host. The third transcript which was up-regulated in “*B. duttonii*-infected” *O. moubata* was annotated as putative lysozyme (A0A2R5LCC0). Lysozymes are antimicrobial peptides which are stimulated by the ingestion of blood. They have already been isolated in the midgut of soft ticks of the genus *Ornithodoros* and their role in bacterial defence has been described [25,26,83,84]. In two other studies by Tanaka et al. and Johns et al. [85,86], increased expression of lysozymes were observed when hard ticks of the species *Haemaphysalis longicornis* and *Dermacentor variabilis* were exposed to bacteria, also suggesting possible roles of lysozyme as innate immunity of ticks against microorganisms. Apart from the endogenous enzymes like lysozymes, it is proposed that Ixodidae [87] and Argasidae [88] apparently can use the hemoglobin of the host as an antimicrobial agent (hemocidins) in the midgut [34]. The fourth transcript was annotated as defensin (A0A1Z5KWQ9). These small proteins are acting as antimicrobial peptides in vertebrates, invertebrates and plants and serve as a defence against microbial pathogens, primarily bacteria, but also fungi and toxins. In a study by Araujo et al. a defensin that showed ~80% amino acid identity with *O. moubata* defensin A was by far the most abundant immunity related transcript in the midgut of *Ornithodoros rostratus* fourth instar nymphs [26]. Interestingly, Nakajima et al. found that synthetic defensin A from *O. moubata* showed antibacterial activity against many Gram-positive bacteria but not Gram-negative bacteria. Since they did not include Gram-negative *Borrelia* species in the antibacterial activity assay, we cannot determine whether defensin expression is induced by infection with *Borrelia duttonii* in our experiments.

Using next-generation sequencing, we obtained a de novo assembly of the midgut transcriptome that significantly increased the genomic resources available for *O. moubata*. Further, our study is the first RNA-seq study analysing infection-induced changes in gene expression in the midgut of a tick species in the family Argasidae. Our results support previous findings and contribute to a better knowledge of the complexities of the tick metabolism and immune system and pave the way for further study and characterization of proteins that modulate blood digestion and microbial infections in the agasid tick *O. moubata*.

## Figures and Tables

**Table 1:** Transcriptome statistics. Raw reads from this study and from the Sequence Read Archive were assembled into contigs, representing the transcriptome. Transcriptomes were then combined and finally, reads from this study were mapped back to the combined contigs.

	This Study	SRA Data	Combination
Number of raw reads	92,373,604	334,384,687	NA
Number of contigs	15,083	202,950	218,033
N50 #	1213 bp, n = 4378	5169 bp, n = 22,677	4917 bp, n = 24,269
Maximum contig length	5729 bp	56,176 bp	56,176 bp
Mean contig length	1065.2 bp	1943.5 bp	1882.7 bp
Mean contig GC	44.6 mol%	47.0 mol%	47.1 mol%
Mean back-mapping rate with Salmon	ND	ND	76.9%

# N50, 50% of the assembly in n sequences; ND: not determined; NA: not applicable.

**Table 2:** Selected genes predicted to be involved in protein metabolism, carbohydrate metabolism and transport, lipid metabolism and transport and response to oxidative stress. It is shown the Fold Change Value. Experimental groups 1 and 2 refer to “engorged vs. unfed—non-infected” and “*Borrelia duttonii*-infected vs. non-infected—engorged”, respectively.

UniProt_ID	Protein Name	Organism	Gene Ontology (GO)	Experimental Group	log2 Fold Change
Protein metabolism and transport					
A0A1Z5KYM0	Xaa-pro aminopeptidase	*Ornithodoros moubata*	metalloaminopeptidase activity [GO:0070006]	1	−10.96
A0A293LQ15	Peptidase A1 domain-containing protein	*Ornithodoros erraticus*	integral component of membrane [GO:0016021]; aspartic-type endopeptidase activity [GO:0004190]	1	5.54
Carbohydrate metabolism and transport					
A0A1Z5L4Q7	Alpha-mannosidase	*Ornithodoros moubata*	alpha-mannosidase activity [GO:0004559]; carbohydrate binding [GO:0030246]; metal ion binding [GO:0046872]; mannose metabolic process [GO:0006013]	1	−3.39
A0A1Z5L0Z3	UDP-N-acetylglucosamine 4-epimerase	*Ornithodoros moubata*	UDP-glucose 4-epimerase activity [GO:0003978]; UDP-N-acetylglucosamine 4-epimerase activity [GO:0003974]; galactose metabolic process [GO:0006012]	2	11.10
A0A2R5LGB3	Putative chitinase	*Ornithodoros turicata*	chitin binding [GO:0008061]; carbohydrate metabolic process [GO:0005975]	2	9.07
Lipid metabolism and transport					
A0A1Z5KX70	Lipoprotein	*Ornithodoros moubata*	lipid transporter activity [GO:0005319]	1	−3.28
A0A1Z5LA57	RING-type domain-containing protein	*Ornithodoros moubata*	metal ion binding [GO:0046872]; NAD+ binding [GO:0070403]; fatty acid metabolic process [GO:0006631]	1	4.80
L7LTX7	Transmembrane protein 188	*Rhipicephalus pulchellus*	cytoplasm [GO:0005737]; integral component of membrane [GO:0016021]; Nem1-Spo7 phosphatase complex [GO:0071595]; nuclear membrane [GO:0031965]; lipid metabolic process [GO:0006629]; positive regulation of protein dephosphorylation [GO:0035307]	1	9.03
Response to oxidative stress					
A0A1D2AJ30	Thioredoxin peroxidase	*Ornithodoros brasiliensis*	peroxidase activity [GO:0004601]; peroxiredoxin activity [GO:0051920]	1	4.28
A0A1Z5LDY2	Thioredoxin-like protein 1	*Ornithodoros moubata*		1	3.41
A0A1Z5LEL5	Cytochrome P450	*Ornithodoros moubata*	heme binding [GO:0020037]; iron ion binding [GO:0005506]; monooxygenase activity [GO:0004497]; oxidoreductase activity, acting on paired donors, with incorporation or reduction of molecular oxygen [GO:0016705]	1	9.74
A0A1Z5LEL5	Cytochrome P450	*Ornithodoros moubata*	heme binding [GO:0020037]; iron ion binding [GO:0005506]; monooxygenase activity [GO:0004497]; oxidoreductase activity, acting on paired donors, with incorporation or reduction of molecular oxygen [GO:0016705]	2	−10.06
A0A2R5L8R9	Putative gamma-glutamyltransferase	*Ornithodoros turicata*	integral component of membrane [GO:0016021]; glutathione hydrolase activity [GO:0036374]; transferase activity [GO:0016740]; glutathione catabolic process [GO:0006751]	2	−11.10
A0A1Z5L521	Uncharacterized protein	*Ornithodoros moubata*	acireductone dioxygenase [iron(II)-requiring] activity [GO:0010309]; methionine biosynthetic process [GO:0009086]	1	−12.99
A0A1Z5KXT5	Sulfotransferase	*Ornithodoros moubata*	sulfotransferase activity [GO:0008146]	1	−11.39
A0A1Z5L722	GDP-4-keto-6-deoxy-D-mannose-3,5-epimerase-4-reductase	*Ornithodoros moubata*	GDP-L-fucose synthase activity [GO:0050577]; translation initiation factor activity [GO:0003743]; ‘de novo’ GDP-L-fucose biosynthetic process [GO:0042351]	1	2.94
A0A2R5L8F7	Gamma-glutamylcyclotransferase	*Ornithodoros turicata*	gamma-glutamylcyclotransferase activity [GO:0003839]; glutathione specific gamma-glutamylcyclotransferase activity [GO:0061928]; glutathione catabolic process [GO:0006751]	1	12.14
A0A1Z5L305	Sulfotransfer_1 domain-containing protein	*Ornithodoros moubata*	sulfotransferase activity [GO:0008146]	2	−4.36

## Data Availability

The raw sequencing data along with deduced Salmon read count tables, de novo assembly and substantial metadata are available at ArrayExpress (http://www.ebi.ac.uk/arrayexpress, accessed on 21 February 2022) under the accession number E-MTAB-11507.

## Supplementary Materials

The following supporting information can be downloaded at: https://www.mdpi.com/article/10.3390/microorganisms10030525/s1, Table S1. Basic summary of analysed samples, treatments and sequencing results. Table S2. Additional sequencing data from *Ornithodoros moubata*, that was included into the de novo assembly. Table S3. List of significantly differentially expressed transcripts. Figure S1. Expression data for selected immune-related transcripts in *Ornithodoros moubata*.
