# Supplementary material for: Early Transcriptional Changes in the Midgut of Ornithodoros moubata after Feeding and Infection with Borrelia duttonii
Source: Microorganisms. 2022 Feb 28;10(3):525. doi: 10.3390/microorganisms10030525 (PMC8948914; doi:10.3390/microorganisms10030525)
Supplement: Supplementary file 1 [file microorganisms-10-00525-s001.zip › Supplemental_Material_TableS1_TableS2_FigureS1.pdf]

Supplementary Materials

Table S1. Basic summary of analysed samples, treatments, and sequencing results. Each sample represents the midgut of an *Ornithodoros moubata* individual

Identifier	Treatment	Infection	Number of trimmed reads (millions)	Mean read length [bp]
lib03352	unfed	non-infected	7.6	156
lib03353	unfed	non-infected	9.4	153
lib03354	unfed	non-infected	7.6	157
lib03355	engorged	non-infected	8.5	158
lib03356	engorged	non-infected	9.3	150
lib03357	engorged	non-infected	11.1	155
lib03358	engorged	*Borrelia duttonii*-infected	9.5	149
lib03359	engorged	*Borrelia duttonii*-infected	8.1	140
lib03360	engorged	*Borrelia duttonii*-infected	11.6	121

Table S2. Additional sequencing data from *Ornithodoros moubata* included into the de novo assembly

SRA run accession	Description	Number of reads (millions)	BioProject accession	Publication
SRR12771191	*Ornithodoros moubata*: sialotranscriptome	51.4	PRJNA667315	Oleaga et al. 2021
SRR12771192	*Ornithodoros moubata*: sialotranscriptome	42.6	PRJNA667315	Oleaga et al. 2021
SRR12771193	*Ornithodoros moubata*: sialotranscriptome	40.9	PRJNA667315	Oleaga et al. 2021
SRR12771194	*Ornithodoros moubata*: sialotranscriptome	45.1	PRJNA667315	Oleaga et al. 2021
SRR12771195	*Ornithodoros moubata*: sialotranscriptome	45.6	PRJNA667315	Oleaga et al. 2021
SRR12771196	*Ornithodoros moubata*: sialotranscriptome	50.0	PRJNA667315	Oleaga et al. 2021
SRR5313934	*Ornithodoros moubata*: midgut genes after blood feeding	26.3	PRJNA377416	Oleaga et al. 2017
SRR5313935	*Ornithodoros moubata*: midgut genes after blood feeding	32.6	PRJNA377416	Oleaga et al. 2017

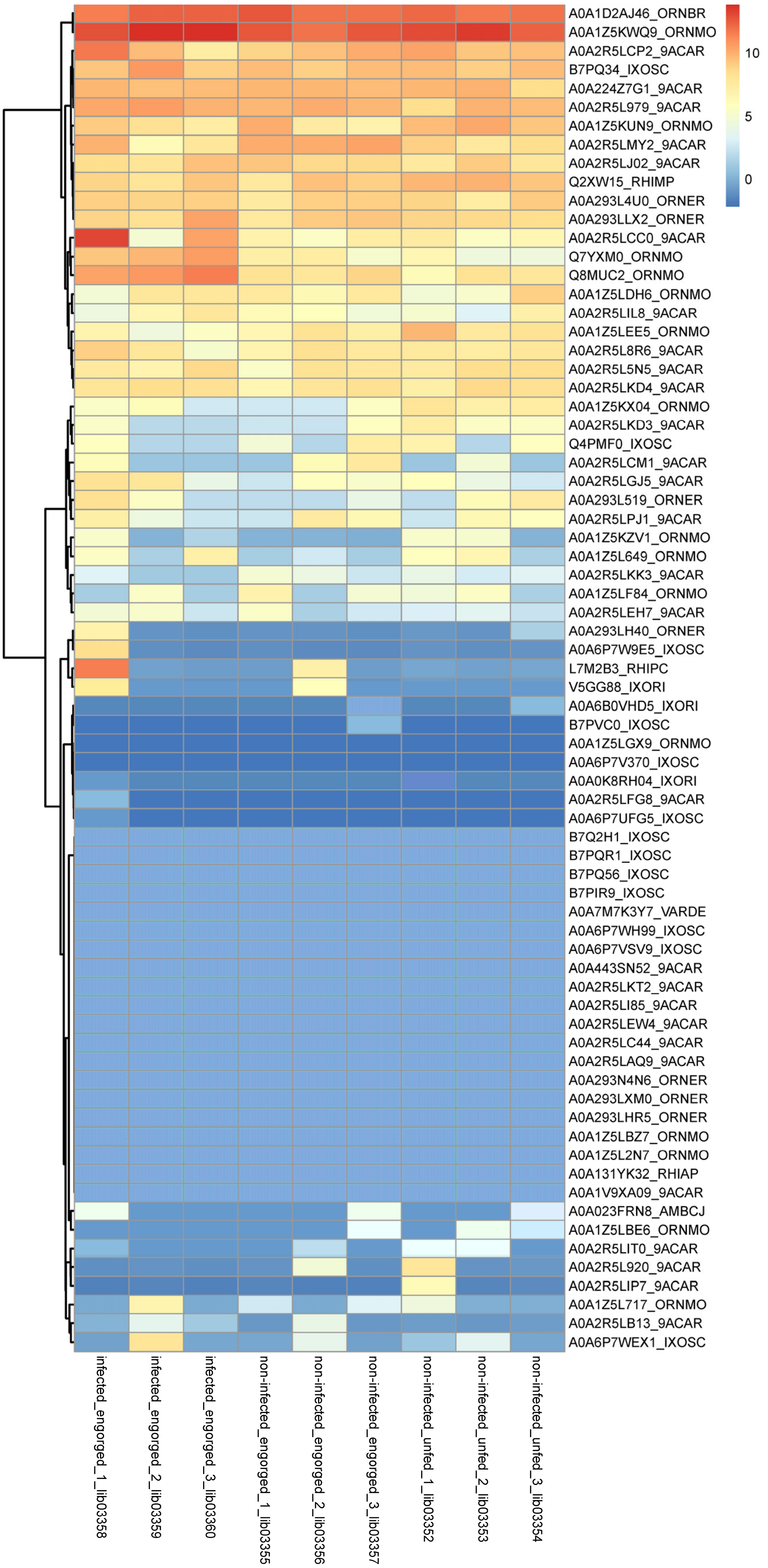

Figure S1. Expression data for selected immune-related transcripts in *Ornithodoros moubata*. The selected transcripts matched one of the following gene ontology terms: defence, dorin, lysozyme, oxidative stress response, antimicrobial peptide, innate immune response. The expression counts are presented as regularized log transformed data.
